# Mental health literacy and psychological outcomes in dual-career athletes

**DOI:** 10.3389/fspor.2025.1609042

**Published:** 2025-08-13

**Authors:** Janja Usenik, Eva Kranjec

**Affiliations:** Faculty of Education, University of Maribor, Maribor, Slovenia

**Keywords:** dual-career athletes, anxiety, depression, mental well-being, mental health literacy

## Abstract

**Introduction:**

Dual-career athletes experience the unique challenges of balancing sporting, academic, and vocational pursuits, which can strain their well-being and increase psychological distress. This study examined the mental health of Slovenian dual-career athletes, including the prevalence of anxiety, depression, and overall well-being, as well as their mental health literacy and its potential relationship with psychological distress and well-being.

**Methods:**

219 Slovenian dual-career athletes (58% female; age 15–29) completed self-report measures of anxiety (GAD-7), depression (PHQ-9), and mental well-being (WHO-5), alongside the Slovenian version of the Mental Health Literacy Scale (S-MHLS). Point and lifetime prevalence of mental health problems were assessed. Multiple regression analyses were employed to examine group differences and predictors of mental health outcomes.

**Results:**

Moderate to severe symptoms of anxiety and depression were reported by 22% and 23% of athletes, respectively, while 43% scored below the threshold for positive mental well-being. Female athletes reported significantly poorer mental health across all indicators. Regression analyses showed that greater knowledge about where and how to seek mental health information predicted lower levels of anxiety and depression and higher well-being. In contrast, more stigmatizing attitudes were linked to higher psychological distress.

**Discussion:**

These findings suggest that improving athletes’ mental health literacy could help protect their well-being in the dual-career context. Tailored strategies to promote help-seeking and reduce stigma, while considering gender and the demands of dual careers, may be crucial for enhancing young athletes’ psychological well-being and creating more supportive environments.

## 1 Introduction

The concept of a dual career refers to athletes pursuing both sports and academic or professional development simultaneously (1). In such a dual career, an athlete's development represents a series of interconnected transitions across multiple domains—athletic, psychological, psychosocial, academic, vocational, and financial as he/she progresses through different athletic career stages (2). These transitions occur simultaneously and influence each other, shaping the athlete's performance and overall well-being. Within this dual career perspective, the athlete is viewed as a whole person with a multidimensional development spanning both athletic and non-athletic domains, providing a comprehensive understanding of the multiple factors influencing their mental health throughout their career and beyond.

A dual career can offer numerous benefits, such as positive identity development, intellectual stimulation, improved life balance, financial security, and career preparation beyond sports (3). However, balancing elite sports with academic development can represent a challenging path that entails additional barriers and demands, such as high expectations in both domains, inadequate recovery, and lack of time, support, or flexibility (3). Athletes may simultaneously struggle with sport-related stress [e.g., performance slumps, injuries, competition pressure, selection dilemmas; (4)], relationship tensions (5), academic pressures [e.g., exams and deadlines; (6)], and financial strain (7). Struggling to manage these pressures can lead to psychological distress, reduced well-being, and even early dropout from sport or education (8).

In the past decade, dual-career athletes’ mental health has emerged as a crucial factor for optimal athletic and academic career development (9). Researchers, practitioners, and policymakers emphasize mental health as a key factor for a successful dual career (10). They also highlight the responsibility of dual career organizations and support providers in protecting and enhancing student-athletes' mental health (11). While mental health has traditionally been defined by the absence of mental illness, recent frameworks, such as Keyes' (12) dual-continuum model, emphasize that mental well-being and mental illness exist on separate but related continuums. This means that an athlete can experience psychological distress (e.g., anxiety, depression) while still maintaining aspects of positive mental health (13). Conversely, an athlete who does not meet clinical diagnostic criteria for mental illness may still struggle with low levels of well-being, motivation, and life satisfaction (14). A mental health continuum approach appreciates that mental health is not a discreet, fixed state of mind, mood, or behaviour, but it is an ever-evolving state of being (15). Positive mental health is commonly conceptualized through indicators of subjective well-being, including emotional vitality, life satisfaction, and positive psychological functioning. A widely used measure of these constructs is the WHO-5 Well-Being Index, which captures the frequency of positive mood states—such as feeling relaxed, energetic, and in good spirits—over the past two weeks (16). In the present study, positive mental health was operationalized using this instrument, offering a complementary perspective to symptom-based assessments of psychological distress, such as anxiety and depression.

While a dual career is believed to help balance the stressors of competitive sport and enhance student-athlete well-being (6), it can also place significant strain on their mental health. Both elite athletes (17) and non-athlete students (18) are at risk for mental health issues, with some suggesting that the dual demands of sport and academics put student-athletes at an even higher risk (19). Research shows that athletes experience a wide range of subclinical and clinical mental health symptoms and disorders (20). Nearly half of dual career athletes experience symptoms of at least one mental health disorder during their careers (21), with prevalence rates for common disorders ranging from 19% to 34% over periods of 4 weeks to 12 months (17). Athletes have been found to experience a wide range of disorders including depression, anxiety, substance abuse, and eating disorders (22). For depression, the point prevalence among athletes varies, with estimates ranging from under 10% (23) to as high as 30%–34% (22, 24). Generalized anxiety disorder prevalence among athletes ranges from 6% for clinically confirmed cases to 14.6% for elevated self-reported symptoms (20). It is important to note that most studies comparing student-athletes to the general youth population find that student-athletes report similar or even lower prevalence of mental health symptoms overall [for a review, see (25)]. Mental health statistics for student-athletes may nevertheless be underestimated, possibly due to their low tendency to seek help (26).

While these mental health challenges affect many dual-career athletes, they do not do so uniformly. Gender differences in mental health symptoms among athletes have been consistently documented across both elite and youth sport populations. Recent meta-analytic evidence shows that female athletes are significantly more likely to report symptoms of anxiety, depression, psychological distress, and disordered eating compared to their male counterparts, who in turn report higher rates of alcohol misuse, substance abuse, and gambling problems (27). Similarly, Saarinen et al. (25), in a comprehensive scoping review of adolescent student-athletes in European sport school contexts, found that although student-athletes may demonstrate lower overall rates of mental illness than the general youth population, female athletes consistently reported higher levels of psychological distress. These patterns have been linked to both biological factors and social mechanisms, including gendered expectations, unequal support structures, and the pressures of pursuing academic and athletic excellence simultaneously (28, 29). Given these disparities, there is growing consensus that gender-sensitive mental health strategies are needed to effectively support the well-being of female student-athletes within dual-career environments.

In athletes, most mental health disorders emerge during adolescence and early adulthood, with the first onset in elite athletes typically occurring around age 19 (21). Symptoms often intensify as the demands of competitive sport increase, particularly during peak performance years (15). Adolescence is a critical period for mental health, as rapid biological, psychological, and social changes increase vulnerability to anxiety, depression, and other mental health disorders (30). Emerging adulthood brings new challenges, including greater independence, academic or career transitions, and financial responsibilities, all of which can contribute to stress and mental health difficulties (31). These two stages of development typically coincides with the peak competitive years for elite athletes (32). In addition to these general developmental challenges, major athletic career transitions, such as the transition from junior to senior levels, cultural adjustments, major competitions, and retirement, pose significant mental health risks. The junior-to-senior transition exposes athletes to increased demands in training, competition, organization, and lifestyle, often coinciding with higher education enrollment (15).

In Slovenia, the dual-career system is shaped by a combination of national legislation and institutional autonomy. During primary education (ages 6 to 14), talented young athletes may already begin training seriously, and schools can offer some basic accommodations, such as schedule adjustments or support for competition absences. These adjustments become more formalized during secondary education (ages 15 to 19), where many student-athletes attend specialized sport schools that provide structured support, including flexible timetables, modified workloads, and coordinated academic planning. These measures are intended to help athletes balance their academic and athletic responsibilities more effectively. However, such support is less consistent beyond the secondary level. In higher education (typically age 19+), universities operate autonomously under the Higher Education Act and determine their own academic calendars and study regimes. Consequently, elite athletes at this stage often rely on informal arrangements or must negotiate academic flexibility individually, often with the support of sports associations or coaches (33). This variability in institutional support across educational stages may influence how athletes experience stress, manage their responsibilities, and access help—factors that are closely tied to mental health and mental health literacy. The Slovenian context, therefore, provides a meaningful backdrop for exploring how MHL relates to psychological outcomes across different phases of the dual-career pathway.Mental health literacy (MHL) refers to the knowledge and attitudes that aid in recognition, management and prevention of mental health issues (34). With the increased interest in athletes' mental health, MHL also became an important topic, especially since research suggests that MHL contributes to positive mental health within the dual career environment (35).

MHL emerged from research on health literacy, which demonstrated a link between low functional literacy and negative health outcomes (36). Health literacy refers to an individual's ability to access, evaluate, and apply health information to make informed decisions about their well-being and healthcare (37). MHL is recognized as a subset of health literacy (34), broadening the scope of health knowledge to include both physical conditions and mental health diagnoses (36). Expanding on the concept of health literacy, Kutcher et al. (34) outlined four key components of MHL: (a) knowledge of mental health management; (b) knowledge of the different types of mental health disorders and treatments; (c) ability and action to decrease mental health stigma; and (d) appropriate help-seeking behaviours, which involves knowing when and where to seek professional care and enhancing self-care practices.

At an individual level, having adequate MHL is associated with better health outcomes, such as suicide prevention (38). In the general student population, lower MHL has been linked to reduced help-seeking behaviours, as students with limited knowledge struggle to recognize clinical symptoms and are uncertain about where to seek professional support (39). Research on athletes' MHL is limited, but existing studies indicate that sufficient levels of MHL in athletes contribute to better recognition and interpretation of behaviour concerning overall well-being (40). Mental health literacy therefore appears to be a critical factor in the well-being of athletes. In dual careers, in which athletes face numerous pressures in multiple domains of their development, MHL can play a crucial role in helping athletes recognize distress, seek appropriate support, and manage mental health proactively (40).

In recent years, several MHL interventions have been developed to support athletes' psychological well-being by reducing stigma and improving help-seeking knowledge. Programs such as Help Out a Mate (41), Ahead of the Game (AOTG) (42, 43) and Team Talk (44) have demonstrated that sport-based, developmentally tailored interventions can enhance athletes' mental health knowledge, increase help-seeking intentions, and build resilience through psychoeducation and team-based discussions. Systematic reviews indicate that these programs are especially effective in reducing stigma and improving mental health literacy outcomes (45–47). However, despite growing recognition of their importance, MHL programs are still not systematically embedded in the curricula of most sports schools. For older athletes, structured MHL training remains scarce, although some elite sport systems have begun integrating athlete welfare or mental health support modules into high-performance programs.Despite promising evidence for existing MHL programs, their applicability across different stages of athletic development remains underexplored. The present study includes both adolescent/pre-elite and elite-level dual-career athletes. While these groups share overlapping dual-career pressures, such as balancing academic demands with training and competition, they may face distinct psychological and contextual challenges. Elite athletes often experience heightened performance expectations, international travel, and public scrutiny, whereas pre-elite athletes more frequently encounter uncertainty regarding future sport careers, transitions between competitive levels, and identity development. By including both groups, this study seeks to capture a more comprehensive picture of how mental health literacy relates to psychological outcomes across the dual-career pathway.

Given the increasing recognition of mental health as a critical component of dual-career athletes' well-being, this study aims to explore the relationship between mental health literacy (MHL) and key mental health indicators, including anxiety, depression, and mental well-being, in dual-career athletes. While previous research has established that dual career athletes face unique psychological challenges due to the dual demands of sport and education and/or vocation, little is known about the relationship between MHL and different mental health aspects. By investigating the interplay between MHL and mental health symptoms, this study seeks to identify whether greater MHL serves as a protective factor in managing psychological distress and promoting well-being among dual-career athletes.

Specifically, this study has three main objectives: (1) to evaluate the prevalence and severity of anxiety, depression, and overall mental well-being within Slovenian dual-career athletes; (2) to assess the levels of their MHL, examining potential differences based on gender and developmental stage (adolescence vs. emerging adulthood); and (3) to investigate the predictive role of MHL in mental health outcomes, determining whether higher MHL is associated with lower levels of anxiety and depression and greater mental well-being.

## 2 Methods

### 2.1 Participants

The sample consisted of 219 Slovenian dual-career athletes, including 127 females (58%) and 92 males (42%). The mean age of the participants was 17.7 years (*SD* = 3.26), with an age range of 15 to 29 years. Most athletes (90.4%) combined their sporting careers with education, while a smaller proportion (9.6%) had both education and work alongside their sporting commitments. Regarding their educational level, younger athletes (aged 15 to 19) were predominantly enrolled in upper secondary schools with student-athlete programs (e.g., sports departments of gymnasiums), whereas older athletes attended higher education institutions. In terms of competition level, most athletes took part in international competitions (51.6%), followed by national competitions (42.5%). A smaller percentage took part in Olympic or Paralympic events (3.2%) or competed at regional level (2.7%). Based on competition level, the sample included both adolescent/pre-elite athletes (those competing primarily at national or youth international levels) and elite-level athletes (those competing at senior international, Olympic, or Paralympic levels).

In terms of sports disciplines, 47.9% of athletes competed mainly in individual sports, while 44.3% competed in team sports. In addition, there was a subgroup of athletes who practiced both sports: 4.1% primarily focused on individual sports but occasionally participated in team competitions, and 3.7% primarily practiced team sports and occasionally participated in individual competitions. In total, the sample comprised 42 different sports, with the eight most common disciplines accounting for 69.7% of the sample. These included football, athletics, basketball, volleyball, swimming, judo, tennis and handball.

### 2.2 Measures

The **Generalized Anxiety Disorder Assessment-7** [GAD-7; (48)] is a 7-item screening tool developed to assess the severity of generalized anxiety disorder. For each item, athletes were asked to rate the severity of their symptoms over the past two weeks (e.g., “Feeling nervous, anxious, or on edge”) using a 4-point Likert scale, ranging from 0 (not at all) to 3 (nearly every day). The total score was calculated by adding up the answers to all seven items, resulting in a possible range of 0 to 21. Higher scores indicate a higher level of generalized anxiety. Cut-off values for clinical severity levels are as follows: 5 ≥ mild, 10 ≥ moderate, 15 ≥ severe. The original version of the GAD-7 has demonstrated strong validity and reliability (Spitzer et al., 2006), as has its Slovenian adaptation on a sample of students [see (49)]. In our sample, a Cronbach's alpha of 0.92 was determined.

The **Patient Health Questionnaire-9** [PHQ-9; (50)] is a self-report measure consisting of nine items designed to assess the severity of depression in the general population. Athletes rated the severity of their symptoms over the past two weeks (e.g., “Feeling down, depressed, or hopeless”) using a 4-point Likert scale, ranging from 0 (not at all) to 3 (nearly every day). The total score is calculated by summing the responses to all nine items, resulting in a possible range from 0 to 27, with higher scores indicating greater levels of depression. Cut-off values for clinical severity levels are as follows: 0–4 none-minimal, 5 ≥ mild, 10 ≥ moderate, 15 ≥ moderate/severe, 20 ≥ severe. The PHQ-9 has been used in the Slovenian healthcare population and has demonstrated high internal reliability [see (51)]. In our sample of dual-career athletes, a confirmatory factor analysis (CFA) was performed using MLR estimator (skewness: 0.28 to 2.06; *Q1* = 0.43, *Q3* = 1.16; kurtosis: −1.19 to 3.47; *Q1* = –0.90, *Q3* = 0.69) to test the one-factor model. The PHQ-9 proved to be a valid measure: RMSEA = 0.05; CFI = 0.98; TLI = 0.97; SRMR = 0.03. The standardized factor loadings ranged from 0.63 to 0.78. Additionally, Cronbach's alpha was 0.89.

The **Well-Being Index** [WHO-5; full version available in (16)] is a self-report tool designed to measure mental well-being. Athletes rated each of the five positively worded items (e.g., “I woke up feeling fresh and rested”) on a 6-point Likert scale, ranging from 0 (at no time) to 5 (all the time). The total raw score is obtained by summing the ratings for all five items, resulting in a score range from 0 to 25. Higher scores indicate better mental well-being. Although the raw score can be converted to a percentage score by multiplying by 4 (16), our study utilized the raw score. The original scale has proven to be valid and reliable measure (16). Furthermore, the Slovenian version of the WHO-5 has been shown to be psychometrically sound in a sample of students [see (52)]. In our sample, a Cronbach's alpha of 0.88 was determined.

The point and lifetime prevalence of mental health problems was assessed using two dichotomous (yes/no) questions, following the approach of previous studies [e.g., (21)]. To assess current mental health problems, participants were asked, “Are you experiencing psychological suffering right now (daily for at least the last two weeks) so severe that you have obvious difficulties functioning as usual in everyday life and/or in sports?” To determine lifetime prevalence, they responded to the question, “Have you ever experienced psychological suffering (daily for at least two weeks) so severe that you had significant difficulties functioning as usual in everyday life and/or in sports?” If participants responded “yes” to the lifetime prevalence question, they were also asked to indicate the age of onset and the number of previous episodes (1, 2 or more than 2).

The **Mental Health Literacy Scale** [MHLS; (53)] assesses various aspects of mental health literacy. In contrast to the original unidimensional scale, which consists of 35 items, the Slovenian version of the MHLS (S-MHLS) (54) comprises 27 items, which are divided into four theoretically and psychometrically valid factors. Different response formats are used for these factors: (1) Attitudes towards people with mental health problems (e.g., “How willing would you be to move next door to someone with a mental illness?”; response scale: 1—definitely unwilling, 5—definitely willing; 7 items); (2) General attitude towards mental health problems and help-seeking (e.g., “Mental illness is not a real medical illness”; response scale: 1—strongly disagree, 5—strongly agree; 8 items); (3) Recognition of mental disorders (i.e., identifying descriptions of mental disorders, such as generalized anxiety disorder, major depressive disorder, and agoraphobia; response scale: 1—very unlikely, 4—very likely; 8 items); (4) Knowledge about seeking mental health information (e.g., “I am confident that I know where to seek information about mental illness”; response scale: 1—strongly disagree, 5—strongly agree; 4 items). The total score for each factor is calculated as the average response. A Slovenian validation study showed reliable internal consistency for all factors, although the factor Knowledge of seeking mental health information showed the lowest reliability [α = .59; (54)]. In the current study, Cronbach's alpha values for the four factors were as follows: Attitude toward people with mental health problems (.86), General attitude toward mental health problems and help-seeking (.82), Recognizing mental disorders (.70), Knowledge of seeking mental health information (.50).

### 2.3 Procedure

The study was approved by the Research Ethics Committee of the University of Maribor (approval no. 038-08-143/2023/9/FFUM). Prior to data collection, a pilot study was conducted to test the comprehensibility of the questionnaires among the athletes. The final questionnaires were distributed using 1KA, an open-source online survey platform. Data collection took place over a three-month period, starting in May 2024, using a multi-channel recruitment approach. Secondary schools with student-athlete programs were contacted, and the National Olympic Committee supported recruitment efforts by sending email invitations to registered dual-career athletes. Eligibility was based on active secondary school enrolment and participation in competitive sports at the regional, national or international level. For underage participants (<18 years), only those with prior parental or guardian consent were included in the study. Participants were given access to the study via a unique link and provided their electronic consent before proceeding. They first read a brief introduction explaining the purpose of the study, the voluntary nature of participation, and the assurance of data confidentiality. By clicking to proceed to the next page, they declared that they were informed about the nature of the study and agreed to the terms of participation. A gentle reminder function was provided to encourage completion of unfinished responses.

### 2.4 Data analyses

First, Mplus 8.0 (55) was used to conduct a confirmatory factor analysis (CFA) for the PHQ-9 measure, as it has not yet been validated in the Slovenian context. The following cut-off values indicated acceptable model fit (56): CFI and TLI ≥ 0.90, RMSEA and SRMR ≤ 0.08. Statistical analyses were conducted using IBM SPSS Statistics (version 29.0). Descriptive statistics, Pearson's correlation analysis, and regression analyses were performed. Group differences in key variables were examined based on gender and developmental stage, with the latter classified according to contemporary frameworks: adolescence (<18 years) and emerging adulthood (18 to late twenties) (57). Independent-samples *t*-tests were employed to assess group differences, with effect sizes interpreted following Cohen's d criteria (small: 0.20; medium: 0.50; large: ≥0.80) (58). To analyse point and lifetime prevalence of mental health problems (MHP) across gender and developmental stage, chi-square tests were conducted. Finally, a series of multiple regression analyses were conducted using the Enter method to investigate the predictive role of mental health literacy in anxiety, depression, and mental well-being. The explanatory power of each regression model was determined based on the following criteria: low (*R*^2^ < 0.13), medium (*R*^2^ = 0.13–0.25), and high (*R*^2^ > 0.26) explanatory power (58).

## 3 Results

Table 1 presents descriptive statistics and group comparisons for anxiety, depression, and mental well-being among dual-career athletes, categorized by gender and developmental stage (adolescence: up to age 18; emerging adulthood/adulthood: ages 18 to late twenties or early thirties). Females reported significantly higher levels of anxiety and depression, as well as lower levels of mental well-being compared to males, with effect sizes ranging from small to medium (Cohen's *d*). No significant differences were observed between adolescents and emerging adults regarding their average levels of anxiety, depression, and mental well-being. A notable proportion of athletes experienced symptoms of anxiety and depression to varying degrees. While many showed none-to-minimal or mild symptoms, over a third of the sample met the criteria for moderate (≥10) to severe symptoms for both anxiety and depression.

**Table 1:** Descriptive statistics and group comparisons for mental health aspects in dual-career athletes.

Variable	Total (*n* = 219)	Males (*n* = 92)	Females (*n* = 127)	Adolescents (*n* = 154)	Emerging Adults/Adults (*n* = 65)	*t* (gender)	*p*	*d*
Anxiety (GAD-7)	8.08 (5.57)	6.43 (5.12)	9.28 (5.60)	8.15 (5.70)	7.92 (5.30)	3.84	<.001	.53
						*t* (DS)	*p*	*d*
% above cut-off					0.27	.784	.04	
10 ≥	21.5	12.0	28.3	23.4	16.9			
15 ≥	14.6	8.7	18.9	14.3	15.4			
Depression (PHQ-9)	8.43 (6.16)	6.96 (5.77)	9.50 (6.24)	8.41 (6.33)	8.48 (5.79)	3.07	.002	.42
						*t* (DS)	*p*	*d*
% above cut-off					−0.07	.941	−.01	
10 ≥	22.8	18.5	26.0	24.0	20.0			
15 ≥	8.7	6.5	10.2	7.8	10.8			
20 ≥	7.3	4.3	9.4	8.4	4.6			
Mental well-being (WHO-5)	13.11 (4.96)	14.46 (5.01)	12.13 (4.71	13.20 (5.10)	12.89 (4.64)	−3.51	<.001	−.48
						*t* (DS)	*p*	*d*
% below cut-off					0.42	.675	.06	
≤13	43.4	33.7	50.4	39.6	52.3			

Note: DS, developmental stage. The series of independent samples *t*-tests were conducted at a significance level *p* < .05.

The results presented in Table 2 show that a notable proportion of dual-career athletes (16.4%) reported experiencing current mental health problems, while the majority (83.6%) did not report such difficulties. No significant differences were found between males and females or between adolescents and emerging adults in point prevalence of mental health problems. In contrast, the lifetime prevalence of mental health problems was higher, with a significantly higher proportion of females than males reporting previous experience of severe mental health problems. In addition, emerging adults were significantly more likely than adolescents to report a history of mental health problems.

**Table 2:** Descriptive statistics and group comparisons for point and lifetime prevalence of mental health problems (MHP) in dual-career athletes.

Variable	Total (*n* = 219)	Males (*n* = 92)	Females (*n* = 127)	Adolescents (*n* = 154)	Emerging Adults/Adults (*n* = 65)	*χ*^2^ (gender)	*p*
Point prevalence of MHP (%)	16.4	14.1	18.1	17.5	13.8	0.62	.433
						*Χ*^2^ (DS)	*p*
						0.45	.501
Lifetime prevalence of MHP (%)	32.1	20.9	40.2	25.5	47.7	9.04	.033
						*χ*^2^ (DS)	*p*
						10.32	.001
	*n* = 70	*n* = 19	*n* = 51	*n* = 39	*n* = 31		
Age of first episode (*M, SD*)	15.61 (3.66)	16.89 (4.07)	15.14 (3.42)	13.90 (2.08)	17.77 (4.09)		
Number of episodes (%)							
1 episode	25.7	26.3	25.5	25.6	25.8		
2 episodes	20.0	10.5	23.5	20.5	19.4		
More than 2 episodes	54.3	63.2	51.0	53.8	54.8		

Note: DS, developmental stage. The series of chi-square tests were conducted at a significance level *p* < .05.

The age of the first episode was determined only for athletes who reported mental health problems. On average, athletes reported experiencing their first episode in early adolescence, while emerging adults reported a slightly later onset compared to adolescents. In addition, more than half of the athletes experienced multiple episodes, and the distribution of recurrence patterns was relatively similar across both genders and developmental groups.

We also examined the average level of mental health literacy aspects in the entire sample and by gender and developmental period (Table 3). Compared to males, females demonstrated significantly more positive attitudes towards people with mental health problems; however, there were no significant differences between adolescents and emerging adults. Similarly, females exhibited slightly greater recognition of mental disorders, although no differences between younger and older athletes were observed.

**Table 3:** Descriptive statistics and group comparisons for mental health literacy subscales (MHLS) in dual-career athletes.

Variable	Total (*n* = 219)	Males (*n* = 92)	Females (*n* = 127)	Adolescents (*n* = 154)	Emerging Adults (*n* = 65)	*t* (gender)	*p*	*d*
	*M* (*SD*)	*M* (*SD*)	*M* (*SD*)	*M* (*SD*)	*M* (*SD*)			
S-MHLS: ATP	25.74 (4.65)	24.90 (5.15)	26.34 (4.16)	25.53 (4.83)	26.22 (4.18)	2.23	.024	.31
						*t* (DS)	*p*	*d*
						−0.99	.322	−.15
S-MHLS: GA	18.42 (5.31)	20.10 (5.95)	17.20 (4.44)	19.23 (5.56)	16.51 (4.13)	−4.12	<.001	−.56
						*t* (DS)	*p*	*d*
						3.55	<.001	.53
S-MHLS: RMD	24.85 (2.96)	24.24 (3.01)	25.30 (2.86)	24.73 (3.12)	25.14 (2.55)	2.65	.004	.36
						*t* (DS)	*p*	*d*
						−0.92	.357	−.14
S-MHLS: KHM	14.73 (2.39)	15.20 (2.40)	14.39 (2.34)	14.71 (2.37)	14.77 (2.46)	−2.50	.013	−.34
						*t* (DS)	*p*	*d*
						−0.17	.863	−.03

Note: ATP, attitudes towards people with mental health problems, GA, general attitude towards mental health problems and help-seeking; RMD, recognition of mental disorders; KHM, knowledge about seeking mental health information; DS, developmental stage. The series of independent samples *t*-tests were conducted at a significance level *p* < .05.

Male athletes reported significantly more negative general attitudes towards mental health problems and help-seeking than female athletes. Compared to adolescents, emerging adults showed significantly fewer stigmatizing attitudes, suggesting a possible change in perception over time. Finally, knowledge about seeking mental health information was significantly higher among males than females, although the effect size was small. No significant differences were found between adolescents and emerging adults. Overall, effect sizes ranged from small to moderate.

A series of multiple regression analyses was conducted to examine the predictive relationships between mental health literacy (MHL) subscales and levels of anxiety, depression, and mental well-being in dual-career athletes (see Table 4). More positive attitudes towards people with mental health problems (i.e., greater acceptance of individuals experiencing mental health illness; ATP), and more negative general attitudes towards mental health problems and help-seeking (i.e., higher stigma; GA), significantly predicted higher levels of both anxiety and depression. Due to the deviation from the expected results, an additional correlation analysis was performed by dividing the sample into two groups: athletes with none or minimal anxiety symptoms (*n* = 66) and those with mild/moderate/severe anxiety symptoms (*n* = 153). Distinct patterns of association emerged between the two groups. Among athletes without anxiety symptoms, a more positive attitude towards individuals with mental health issues was linked to lower levels of anxiety (*r* = –.12, n. s.). In contrast, among athletes experiencing anxiety symptoms, a more positive attitude towards people with mental health conditions was associated with higher levels of anxiety (*r* = .24***). The direction of associations with depression remained consistent across both groups, as observed in the full sample. Although both ATP and GA were associated with lower mental well-being in the Slovenian sample of athletes, these associations did not reach statistical significance.

**Table 4:** Results of multiple regression analyses for prediction of anxiety, depression, and mental well-being from mental health literacy subscales in dual-career athletes.

Predictor variables	Anxiety (GAD-7)	Depression (PHQ-9)	Mental well-being (WHO-5)
	β	β	β
Attitudes towards people with mental health problems (ATP)	.221***	.293***	−.116
General attitudes towards mental health problems and help-seeking (GA)	.231***	.261***	−.027
Recognition of mental disorders (RMD)	.072	.134*	.047
Knowledge about seeking mental health information (KHM)	−.170*	−.138*	.262***
*R*²	.101	.149	.073
*F*	6.04***	9.34***	4.21**

* *p* < .05.

** *p* < .01.

*** *p* < .001.

Furthermore, greater recognition of mental health disorders (RMD) was found to significantly predict increased depressive symptoms, but not anxiety or overall mental well-being. Additionally, a correlation analysis revealed differing patterns in these predictive relationships. Among athletes without depressive symptoms (*n* = 68), a better recognition of mental health disorders was negatively associated with depression (*r* = –.09, n. s.). In contrast, among athletes who reported mild, moderate, or severe depressive symptoms (*n* = 151), this association was positive (*r* = 0.16*).

Finally, greater knowledge about seeking mental health information (KHM) was linked to lower levels of anxiety and depression, as well as higher mental well-being. The regression models demonstrated low to moderate explanatory power.

## 4 Discussion

The present study aimed to investigate the mental health and mental health literacy (MHL) of Slovenian dual-career athletes by addressing three key objectives. First, it examined the prevalence and severity of anxiety, depression, and overall mental well-being in this population. Second, it assessed athletes' levels of MHL, exploring potential differences based on gender and developmental stage (adolescence vs. emerging adulthood). Third, the study explored the predictive role of MHL in mental health outcomes, with the goal of determining whether higher MHL is linked to lower levels of psychological distress and higher levels of well-being.

Findings revealed that a substantial proportion of dual-career athletes reported symptoms of psychological distress. Specifically, 21.5% met the threshold for moderate to severe anxiety, and 22.8% reported symptoms of moderate to severe depression. These rates are in line with previous research on student-athletes, suggesting that psychological distress is a relevant concern in this population, even if not necessarily higher than in general youth samples (14, 25). In their recent study about student-athletes mental health, Moore et al. (59) found that around 15% met the criteria for moderate to moderately severe depression, while 25.8% met the threshold score for generalized anxiety disorder. In the broader athlete population, comparable studies reflect considerable variation. While Weber et al. (23) reported that fewer than 10% of youth athletes showed elevated depressive symptoms, other studies have found substantially higher rates. For example, Poucher et al. (22) reported depression point prevalence as high as 30%–34% in certain athlete samples. Similarly, Reardon et al. (20) noted that the point prevalence of generalized anxiety disorder in athletes ranged from 6% (clinically diagnosed) to 14.6% (self-reported symptoms). Taken together, these findings suggest that the prevalence of anxiety and depression among Slovenian dual-career athletes falls within the range observed in other athletic populations and highlight the importance of monitoring and supporting their mental health. Rather than indicating a universally heightened risk, the results point to the need for context-sensitive mental health support systems that address the specific challenges dual-career athletes face.

In terms of mental well-being, 43.4% of athletes in our study scored below the mental well-being threshold, indicating low positive mental health. In comparison with the rates we found for anxiety and depression, this paints an even more concerning picture of dual-career athletes' overall psychological functioning. The fact that a greater proportion of participants reported low mental well-being than those who met the criteria for psychological distress aligns with the two-dimensional understanding of mental health proposed by Keyes (12). In his dual-continuum model, mental well-being and mental illness are conceptualized as separate but interrelated constructs. This means that an athlete can experience psychological distress (e.g., anxiety, depression) while still maintaining certain elements of positive mental health (13), and vice versa—an athlete may exhibit low well-being even in the absence of clinical symptoms, as was the case for a substantial part of our sample. This supports the notion that using a dimensional approach enables a more nuanced understanding of athletes' mental health, moving beyond binary diagnostic thresholds to better capture the complexity of their psychological experiences (15).

These findings resonate with prior research emphasizing the importance of considering well-being as a distinct and essential component of mental health in dual-career athletes. Aalto et al. (60) highlighted several psychological and social factors—such as perfectionism, elevated stress, low autonomy, and unsupportive environments—that are strongly associated with lower mental well-being in this population. Dual-career athletes often navigate overlapping demands across sport, education, and personal life, which can lead to chronic overload, compromised recovery, and a reduced sense of vitality and balance (3). Even when athletes manage to avoid significant psychological distress, these cumulative stressors may still diminish their capacity to experience emotional, psychological, and social well-being.

In our study, gender differences were evident across all three indicators of mental health. Female dual-career athletes reported significantly higher levels of anxiety and depression, as well as lower levels of mental well-being compared to their male counterparts. These findings are consistent with previous research in both elite and student-athlete populations. For instance, studies have repeatedly shown that female athletes tend to report more internalizing symptoms, such as anxiety and depression (61), while male athletes are more likely to underreport symptoms due to prevailing norms around masculinity and emotional control (22). Within the dual-career context, gendered experiences may further exacerbate this discrepancy. Research suggests that female student-athletes are more likely to pursue a stable dual-career pathway, placing relatively equal emphasis on both academic and athletic commitments (62). This increased striving for excellence in multiple life domains may amplify stress exposure and the risk for emotional exhaustion, particularly in environments that lack adequate support or flexibility, and underscores the importance of gender-sensitive, developmentally appropriate mental health promotion strategies that extend beyond illness prevention and address broader psychosocial well-being (25).

While gender differences were clearly observed, our findings did not reveal significant differences in anxiety, depression, or mental well-being between adolescent and emerging adult athletes. This is somewhat surprising given that previous literature highlights emerging adulthood as a particularly sensitive developmental period for mental health challenges. According to Åkesdotter et al. (21), the typical age for the onset of mental disorders in elite athletes is around 19 years, coinciding with the transition into emerging adulthood. This stage is marked by increased demands for autonomy, identity exploration, and major life transitions—including entry into higher education or more competitive sport levels—which are known to heighten psychological vulnerability (63). Despite these findings, our results suggest that psychological distress and low well-being are already present at comparable levels during adolescence in the dual-career athletes population, possibly due to the early onset of dual-career stressors such as performance pressure, academic workload, and time conflicts.

Moreover, it is important to consider that both adolescents and emerging adults within dual careers navigate multiple simultaneous transitions across athletic, academic, and personal domains (2). As highlighted by Stambulova and Wylleman (3), the dual-career pathway often exposes young athletes to complex demands at an earlier age than their non-athlete peers, potentially blurring developmental distinctions in mental health profiles. While our findings did not indicate statistically significant differences between the age groups, emerging adults did report a slightly higher lifetime prevalence of mental health problems, aligning with broader trends in developmental psychology Auerbach et al. (18). These patterns underscore the importance of early, sustained mental health support across developmental stages and the need for age-appropriate interventions that address both shared and unique challenges faced by adolescent and emerging adult dual-career athletes.

The present study also explored the role of mental health literacy (MHL) in dual-career athletes using the Slovenian version of the Mental Health Literacy Scale [S-MHLS; (42)], which comprises four distinct subscales: (1) Attitudes Towards People With Mental Health Problems, (2) General Attitudes Towards Mental Health Problems and Help-Seeking, (3) Recognition of Mental Health Disorders, and (4) Knowledge About Seeking Mental Health Information. Significant gender differences emerged across all four subscales, with female athletes reporting more positive attitudes toward people with mental health problems, less stigmatizing general attitudes toward mental health and help-seeking, and greater recognition of mental disorders. These results align with earlier studies showing that male athletes tend to hold more stigmatizing beliefs and report lower MHL levels compared to female athletes (39). These gendered patterns are often attributed to prevailing cultural norms around masculinity, emotional control, and help-seeking in sport, particularly in male-dominated or individual sport settings (64). In contrast, male athletes in our sample scored significantly higher on knowledge about where to seek mental health information, which may reflect greater familiarity with navigating systems or online resources, rather than a readiness to apply that knowledge in real-life help-seeking situations.

With respect to the developmental stages, fewer differences were observed. Emerging adults reported significantly more positive general attitudes toward mental health and help-seeking than adolescents, indicating a potential developmental shift in stigma-related beliefs as athletes mature and gain more exposure to mental health education or personal experiences with distress (18). However, no significant differences were found between adolescents and emerging adults in attitudes toward people with mental illness, recognition of mental disorders, or knowledge about seeking help. This suggests that foundational knowledge and beliefs about mental health may be established earlier in adolescence and remain relatively stable into emerging adulthood, or that current interventions in sport and education are beginning to close developmental gaps in MHL. The relatively uniform MHL across age groups in our sample may therefore reflect recent efforts to integrate mental health awareness into youth sport and educational settings, although further targeted interventions are still needed to address stigma and encourage help-seeking. Nevertheless, the higher lifetime prevalence of mental health problems among emerging adults, as revealed in our data, underscores the importance of ongoing MHL efforts tailored to developmental transitions and their associated stressors (21).

In line with the study's third objective, our findings provide important insights into the predictive value of mental health literacy (MHL) for psychological outcomes among dual-career athletes. Of the four MHL subscales, knowledge about seeking mental health information was the most consistent protective factor, predicting lower levels of anxiety and depression and higher levels of mental well-being. This suggests that athletes who know where and how to seek help—whether online, in person, or through formal mental health services—may be better equipped to manage psychological challenges when they arise. These findings align with prior studies indicating that limited knowledge of available mental health resources is a key barrier to timely help-seeking among students and athletes alike (65). In high-demand environments such as dual careers, where athletes must balance sport and education, improving access to such knowledge appears particularly vital.

Interestingly, both more positive attitudes toward people with mental health problems and more negative general attitudes toward mental health problems and help-seeking significantly predicted higher levels of anxiety and depression in dual-career athletes. While this combination may seem contradictory at first glance, it can be understood by considering the distinct nature of each subscale. More accepting attitudes toward individuals with mental illness—reflecting one's willingness to interact with or support people facing psychological challenges—may signal increased empathy or personal relevance, particularly among athletes who are themselves experiencing distress. This is consistent with previous research showing that individuals with lived mental health experience are often less stigmatizing toward others (66). However, such accepting views do not necessarily protect against poor mental health outcomes, suggesting that the directionality between attitudes, personal experience, and well-being is complex. In contrast, more negative general attitudes toward mental health and help-seeking—reflecting internalized stigma—were also linked to greater psychological distress and lower well-being. This subscale includes both stigmatizing beliefs (e.g., “Mental illness is a sign of weakness”) and negative help-seeking attitudes (e.g., “Seeing a professional means you are not strong enough”; “If I had a mental illness, I would not seek help”). These findings align with evidence that stigma, especially when internalized, serves as a powerful barrier to recognizing symptoms, accessing care, and engaging in adaptive coping strategies (67). For dual-career athletes—who often face high expectations in both academic and athletic settings—internalized stigma may further inhibit openness about distress and contribute to avoidance of professional support. As shown in prior research, adolescents and young adults with stigmatized views are less likely to disclose emotional struggles or seek help in a timely manner, leading to poorer long-term outcomes (68). The combination of self-stigma and role conflict—where athletes feel pressure to appear strong, composed, and successful—can result in suppressed emotional expression and delayed intervention, thereby exacerbating psychological symptoms. These findings highlight the importance of addressing not only public stigma but also deeply held beliefs about mental illness and help-seeking within performance-oriented environments such as dual-career pathways.

Additionally, recognition of mental disorders (RMD) was found to significantly predict higher levels of depressive symptoms, though this relationship appears to be influenced by the dual-career athletes' current mental health status. A closer examination of the data revealed that among athletes without depressive symptoms, RMD was not significantly associated with depression. However, among those reporting mild to severe depressive symptoms, higher recognition was positively correlated with greater symptom severity. This suggests that recognition of mental health disorders may function differently depending on whether a dual-career athlete is currently experiencing distress. For those already struggling with depressive symptoms, increased recognition might reflect heightened awareness or focus on psychological challenges, which could potentially amplify distress when not accompanied by effective coping strategies or access to support. In contrast, among athletes without symptoms, recognition alone may not have a meaningful impact—either protective or detrimental.

These findings underscore that while symptom recognition is an important dimension of mental health literacy, its effects may be context-dependent and not inherently protective. In the case of dual-career athletes, who often manage overlapping academic and athletic demands with limited time and support, recognition of symptoms without the confidence, skills, or resources to act may increase psychological burden. This highlights the importance of integrating symptom recognition with other facets of MHL—such as stigma reduction, help-seeking efficacy, and mental health self-management—in order to provide dual-career athletes with the tools they need to effectively respond to distress. In this regard, MHL should not be approached as a checklist of separate components, but as a dynamic and interconnected set of competencies that can support well-being and early intervention in the unique context of dual careers.

The findings of this study carry important implications for mental health support, education, and policy in the context of dual careers. First, the high prevalence of anxiety, depression, and low mental well-being among Slovenian dual-career athletes underscores the urgent need to prioritize mental health within sport and educational systems. Given the early emergence and persistence of psychological distress in this population, mental health initiatives should be implemented proactively, starting in adolescence and continuing through emerging adulthood. These initiatives should be embedded in the environments athletes already navigate—schools, universities, sports clubs, and national sports federations—ensuring accessibility, continuity, and cultural relevance.

Second, the multidimensional nature of mental health literacy (MHL) identified in this study suggests that interventions should move beyond simple awareness campaigns and instead target the full range of MHL competencies. These should include stigma reduction—both public and internalized—accurate recognition of mental health symptoms, and most importantly, the ability and confidence to seek appropriate support. In our sample, knowledge about where and how to access mental health information emerged as the most consistent predictor of lower anxiety and depression and higher well-being. This finding aligns with outcomes from established MHL interventions in sport, such as Help Out a Mate (41), Ahead of the Game (43) and Team Talk (44). These programs demonstrate that sport-integrated, developmentally appropriate interventions can improve mental health knowledge, foster help-seeking intentions, and build psychological resilience. However, as systematic reviews reveal, the impact of such programs on actual help-seeking behaviors remains limited (45–47), echoing our own observation that knowledge alone is insufficient when stigmatizing beliefs persist. Therefore, future interventions must combine practical skill-building with efforts to change underlying attitudes and norms, especially within sport cultures that often valorize toughness and self-reliance.

Third, the gendered patterns observed in both mental health and MHL suggest a need for gender-sensitive approaches. Female athletes reported greater psychological distress and lower well-being, yet also demonstrated more open and empathetic attitudes toward mental health. Meanwhile, male athletes, despite higher knowledge about seeking help, held more stigmatizing beliefs and may be less inclined to act on their knowledge. This highlights the importance of tailoring interventions to address these specific dynamics—normalizing help-seeking for male athletes and supporting emotional coping strategies for female athletes in high-pressure environments. These dynamics are consistent with broader findings across European sport school contexts, where female student-athletes report significantly higher rates of psychological distress, burnout, eating disorders, and dysfunctional sleep patterns than their male peers (25, 29). Female athletes often face unique psychosocial stressors, including body image pressures, lower perceived coaching support, and higher social evaluative concerns, all of which can contribute to compromised mental health (59). In contrast, male athletes may be less likely to acknowledge distress due to internalized stigma or fear of appearing weak, which can contribute to underreporting of symptoms rather than actual lower prevalence (69). Yet most existing interventions—such as Help Out a Mate, Ahead of the Game—were originally designed for male adolescent athletes, and few have been meaningfully adapted to meet the specific needs and experiences of female athletes. For MHL programs to be effective, they must explicitly recognize and respond to these gender-specific stressors. Interventions should be tailored not only to the general demands of dual-career pathways but also to the gender-specific stressors and barriers that influence how male and female athletes experience, express, and manage their mental health. Doing so can help ensure that all athletes—regardless of gender—receive the type and level of support needed to maintain psychological well-being and performance sustainability.

Fourth, the findings suggest that dual-career athletes may benefit from integrated support systems that bridge sport and education. The increasing recognition of MHL underscores the opportunity for more systematic integration into sport school curricula, which remains limited in many settings, including Slovenia. For older athletes, structured MHL training remains scarce, although some elite sport systems have begun integrating athlete welfare or mental health support modules into high-performance programs. To address these disparities, we propose embedding MHL education within sport schools and club environments and expanding the target audience beyond athletes. Coaches, teachers, school counselors, and sport psychologists should work collaboratively to monitor well-being, identify at-risk individuals, and provide timely support. Training key figures in athletes’ networks in mental health literacy—particularly in recognizing early signs of distress and initiating supportive conversations—can strengthen this system of care. Existing evidence suggests that even brief training for coaches and support staff can lead to significant improvements in mental health knowledge and reductions in stigma (70).

These considerations are particularly relevant in the Slovenian context, where the level of formal dual-career support differs across educational stages. While secondary-level sport schools may offer institutional adjustments for athletes, higher education institutions are not uniformly equipped to accommodate the needs of student-athletes. As such, older athletes in our sample may face greater barriers to academic flexibility, time management, and access to mental health resources. We recommend that Slovenian sports schools and universities consider embedding mental health literacy into both academic and athletic development programs. This could involve co-created MHL initiatives delivered via workshops, athlete mentoring, or peer-led formats, supported by trained staff across sport and education sectors. Ensuring consistent access to such programs throughout an athlete's dual-career pathway may help reduce stigma, support early intervention, and promote sustainable well-being.

Finally, national and institutional policy should reflect the recognition that mental health is essential to athletes’ development and performance. Dual-career programs should explicitly incorporate mental health objectives, allocate resources for psychological support, and evaluate their environments using well-being indicators, not just academic or sporting success. Doing so would affirm mental health as a shared responsibility—an integral component of athlete development and not an individual burden to be silently carried.

### 4.1 Limitations and further directions

While this study offers important insights into the mental health and MHL of Slovenian dual-career athletes, several limitations should be noted. Although the sample included athletes from a range of sports and competition levels, the study was conducted within a single national context. The structure of dual-career pathways in Slovenia—marked by a separation between educational and sport systems, limited institutional support, and specific cultural norms regarding mental health—may not reflect the experiences of dual-career athletes in other countries. Future research should adopt cross-national comparative designs to explore how different dual-career models and support systems influence athletes' mental health and literacy.

An additional limitation is the heterogeneity of the sample in terms of athletic level. While the inclusion of both pre-elite and elite-level dual-career athletes reflects the diversity of real-world sport pathways, these groups may differ in their exposure to performance stress, institutional support, and mental health risks. Although our developmental subgroup analyses captured age-related differences, future research would benefit from explicitly stratifying participants by athletic level to better understand how elite status interacts with mental health and literacy outcomes. Tailored interventions may be needed to address the unique challenges each group encounters.

Another limitation concerns the internal reliability of the Knowledge About Seeking Mental Health Information subscale, which was relatively low (Cronbach's α = .50). Although this subscale still emerged as the most consistent protective predictor of mental health outcomes, its psychometric limitations warrant caution in interpretation. Future studies should aim to refine and validate this subscale—especially in athlete populations—to ensure accurate measurement of this crucial aspect of MHL. In addition, the current study did not consider several potentially influential factors, such as the type of sport (e.g., team vs. individual), level of competition, injury history, or access to formal mental health services. Prior research suggests that these variables may meaningfully shape both mental health risk and protective processes in athletic populations (17, 20). Expanding future analyses to include these contextual and sport-specific variables would enable a more nuanced understanding of the diverse experiences of dual-career athletes.

Finally, reliance on self-report measures may introduce reporting biases—particularly underreporting due to stigma or social desirability, which remain salient concerns in high-performance sport environments. While the use of validated instruments adds strength to the methodology, future research would benefit from a multi-method approach. Integrating qualitative data, observational methods, or input from coaches, psychologists, and educators could offer richer insights into how athletes experience, interpret, and manage mental health challenges in dual-career settings.

Despite these limitations, the present study provides a valuable foundation for further exploration of mental health and MHL in dual-career athletes. Future research should prioritize longitudinal studies, evaluate the impact of targeted MHL interventions, and adopt ecological frameworks that take into account the complex interactions between personal, sport-related, and systemic factors. Such efforts are essential for shaping effective, evidence-based strategies to support the mental health and holistic development of athletes navigating the dual-career pathway.

## 5 Conclusion

This study provides novel and timely insights into the mental health and mental health literacy of dual-career athletes in Slovenia. The findings revealed considerable levels of psychological distress and low mental well-being, particularly among female athletes, highlighting the mental health vulnerability of this population. Importantly, the study demonstrated that mental health literacy—especially knowledge about where to seek help—plays a meaningful role in predicting mental health outcomes, offering a promising pathway for targeted interventions. At the same time, the results underscore the complexity of MHL, showing that not all components operate uniformly and that attitudes and recognition may interact with symptom experience in nuanced ways.

Taken together, the findings advocate for a multidimensional and developmentally sensitive approach to promoting mental health within dual-career systems. Strengthening athletes' mental health literacy—by reducing stigma, increasing access to information, and fostering supportive environments—should be recognized as a key pillar of athlete development. As sport and education systems continue to evolve, ensuring that psychological well-being is integrated into dual-career support structures will be essential for helping athletes thrive both on and off the field.

## Data Availability

The raw data supporting the conclusions of this article will be made available by the authors, without undue reservation.
